# Comparing deep learning and pathologist quantification of cell-level PD-L1 expression in non-small cell lung cancer whole-slide images

**DOI:** 10.1038/s41598-024-57067-1

**Published:** 2024-03-26

**Authors:** Leander van Eekelen, Joey Spronck, Monika Looijen-Salamon, Shoko Vos, Enrico Munari, Ilaria Girolami, Albino Eccher, Balazs Acs, Ceren Boyaci, Gabriel Silva de Souza, Muradije Demirel-Andishmand, Luca Dulce Meesters, Daan Zegers, Lieke van der Woude, Willemijn Theelen, Michel van den Heuvel, Katrien Grünberg, Bram van Ginneken, Jeroen van der Laak, Francesco Ciompi

**Affiliations:** 1 Department of Pathology, Radboud University Medical Center, P.O.Box 9101, 6500 HB Nijmegen, The Netherlands; 2 Department of Radiology, Radboud University Medical Center, Nijmegen, The Netherlands; 3 Pathology Unit, Department of Molecular and Translational Medicine, University of Brescia, Brescia, Italy; 4 Department of Pathology, Provincial Hospital of Bolzano (SABES-ASDAA), Bolzano-Bozen, Italy; 5 Department of Pathology and Diagnostics, University and Hospital Trust of Verona, Verona, Italy; 6 Department of Clinical Pathology and Cancer Diagnostics, Karolinska University Hospital, Stockholm, Sweden; 7 Department of Thoracic Oncology, Netherlands Cancer Institute, Amsterdam, The Netherlands; 8 Respiratory Diseases Department, Radboud University Medical Center, Nijmegen, The Netherlands

**Keywords:** Non-small-cell lung cancer, Machine learning

## Abstract

Programmed death-ligand 1 (PD-L1) expression is currently used in the clinic to assess eligibility for immune-checkpoint inhibitors via the tumor proportion score (TPS), but its efficacy is limited by high interobserver variability. Multiple papers have presented systems for the automatic quantification of TPS, but none report on the task of determining cell-level PD-L1 expression and often reserve their evaluation to a single PD-L1 monoclonal antibody or clinical center. In this paper, we report on a deep learning algorithm for detecting PD-L1 negative and positive tumor cells at a cellular level and evaluate it on a cell-level reference standard established by six readers on a multi-centric, multi PD-L1 assay dataset. This reference standard also provides for the first time a benchmark for computer vision algorithms. In addition, in line with other papers, we also evaluate our algorithm at slide-level by measuring the agreement between the algorithm and six pathologists on TPS quantification. We find a moderately low interobserver agreement at cell-level level (mean reader-reader F1 score = 0.68) which our algorithm sits slightly under (mean reader-AI F1 score = 0.55)**,** especially for cases from the clinical center not included in the training set. Despite this, we find good AI-pathologist agreement on quantifying TPS compared to the interobserver agreement (mean reader-reader Cohen’s kappa = 0.54, 95% CI 0.26–0.81, mean reader-AI kappa = 0.49, 95% CI 0.27—0.72). In conclusion, our deep learning algorithm demonstrates promise in detecting PD-L1 expression at a cellular level and exhibits favorable agreement with pathologists in quantifying the tumor proportion score (TPS). We publicly release our models for use via the Grand-Challenge platform.

## Introduction

Immunotherapy with immune checkpoint inhibitors (ICIs) has made a substantial impact in recent years on the treatment of a subset of non-small cell lung cancer (NSCLC) patients^1^, with clinical trials even reporting up to a doubling of median overall survival of patients compared to chemotherapy^2^. However, only a minority of NSCLC patients respond to ICI and only a few have a durable response^3^.

Selecting patients eligible for ICI therapy targeting the interaction between programmed death-ligand 1 (PD-L1) and PD-1 is typically performed based on PD-L1 immunohistochemistry (IHC). PD-L1 expression is estimated visually by a pathologist and expressed as the tumor proportion score (TPS): the percentage of PD-L1 positive (viable) tumor cells as compared to the total number of tumor cells. TPS is currently the only approved biomarker to select NSCLC patients for ICI treatment^3^.

Despite its daily use in the clinic, IHC-based TPS suffers from several fundamental limitations, caused by variations in PD-L1 monoclonal antibodies and staining intensity, heterogeneous PD-L1 expression throughout tumors^4,5^, and the intrinsic limitation of the human visual capability to detect, classify and quantify thousands of tumor cells in histopathology slides^6^. As a result, studies have found substantial interobserver variability at TPS assessment^7,8^. Lastly, TPS has been shown to have an intrinsically limited predictive power as a biomarker: Lu et al.^9^ found an AUC of 0.65 in a meta-analysis of studies assessing the accuracy of PD-L1 IHC predicting response to PD-L1 targeting ICIs.

TPS quantification in NSCLC can be viewed as a combination of three different tasks: (1) localization of cells within the image, (2) classification of individual cells into PD-L1 negative tumor cells, PD-L1 positive tumor cells or ‘other’ (i.e. ‘to be excluded’) and (3) finally calculating a ratio of the positive versus the total number of tumor cells. This combination of tasks makes TPS an excellent candidate for automation by deep learning (DL), as previous works have shown good performance of DL-based computer models for cell localization and classification^10,11^. In addition, addressing TPS quantification by automating localization and classification at cellular level can enable development of novel biomarkers based on spatial analysis of the tumor tissue.

There are a number of prerequisites for developing deep learning systems that detect PD-L1 expression at a cellular level. Firstly, an efficient and scalable way of generating manual annotations is required for training supervised deep learning models. Secondly, cases for the development and validation of the system should be reflective of data in clinical practice. The data should ideally cover a broad range of tissue morphologies, specimen types (biopsy and resections) and scanners. Particular focus should be put on including a wide selection of PD-L1 monoclonal antibodies used in the clinic, as the system should generalize across multiple antibodies and staining expressions. Most technical studies have only reported results within well-controlled, single-center/single-antibody environments^12–14^, which hampers their generalizability and applicability. Lastly, since cell-level DL methods are trained with supervision, a human benchmark based on the input of multiple readers is needed, to establish the theoretical upper bound performance of computer methods. Previous works have only correlated algorithms to TPS as visually estimated by pathologists^12–17^, but to the best of our knowledge, no work exists that quantifies the interobserver variability of pathologists at classifying cell-level PD-L1 expression and compares deep learning methods to cell-level annotations in a multi-reader setting.

In this study, we developed a DL-based PD-L1 detector for NSCLC whole-slide images, trained on multi-centric, multi-PD-L1 assay data. First, we introduce a fast and scalable procedure to generate annotations by developing a nuclei detector that enables semi-automatic multi-class annotations. Second, we used the annotations to develop a multi-class cell detector in PD-L1 IHC. Third, we set up a reader study where a panel of pathologists established a cell-level reference standard. We used this to measure, for the first time in the literature, the interobserver variability of pathologists at determining cell-level PD-L1 expression, providing a benchmark for computer vision algorithms. Finally, in line with previous studies, we correlate the automated TPS derived from the output of our PD-L1 detector to the slide-level visual estimates of pathologists.

## Materials and methods

### Datasets

To capture the wide diversity of PD-L1 IHC in the clinic, we created an international, multi-centric, multi-scanner and multi PD-L1 assay collection of slides that included biopsies, resections and tissue microarrays (TMAs). In total 152 NSCLC patients were included. All patients had a single slide which originated either from the primary tumor or a metastasis to another site in the lungs. Patients originated from one of three clinical centers: (1) 73 patients from the Radboud University Medical Center, Nijmegen, the Netherlands, referred to as *RUMC*, (2) 54 patients from the Sacro Cuore Don Calabria Hospital of Negrar, Verona, Italy, referred to as *NEG* and (3) 25 patients from the Netherlands Cancer Institute-Antoni van Leeuwenhoek Hospital, Amsterdam, the Netherlands, referred to as *NKI.* Material of patients in the *RUMC* and *NEG* cohorts (previously used and described in^18^ and^19^) was gathered as part of routine clinical care. *NKI* patient material belongs to a subset of patients participating in the phase 2 PEMBRO-RT randomized control trial^20^ and was collected pre-treatment. Slides were stained for PD-L1 and scanned by the laboratories of their respective clinical centers. Table 1 shows the scanners, collection period and monoclonal antibodies per dataset, in addition to the distribution of specimen types (see Supplementary materials & methods for additional details).

**Table 1:**
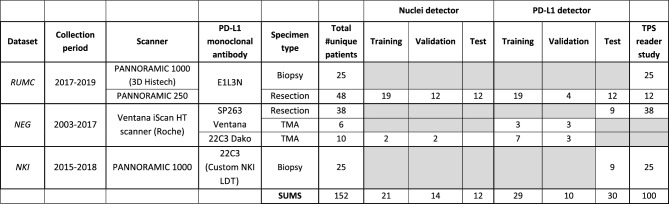
A general overview of the datasets and the number of cases used for each experiment.

For the RUMC dataset, the need for informed consent was waived by the institutional review board of the Radboud UMC, Nijmegen, the Netherlands (reference 2018-4764). For the NEG dataset, the need for informed consent was waived by the Ethic Committee for Clinical Research of the Provinces of Verona and Rovigo (reference 25046). For the NKI dataset, the PEMBRO-RT clinical trial (reference 2014-005118-49) from which cases were taken was approved by the institutional review board of the Netherlands Cancer Institute–Antoni van Leeuwenhoek Hospital, Amsterdam, the Netherlands. The research was performed in accordance with the principles expressed in the Declaration of Helsinki.

The annotations used to train and validate the algorithms were made by trained research assistants (L.M, M.D.A) and one physician (G.S) under supervision of two pathologists (E.M and M.L.S). The pathologists selected regions of interest (ROIs), 3 per slide on average (sized 300 by 300 μm), deemed to be a representative sample of morphologies and cell types typically found in NSCLC histopathology, as well as with heterogeneous distribution of PD-L1 positivity. For the nuclei detector, all nuclei within the ROI were manually point annotated, while for the PD-L1 detector hand-drawn polygons were made for the semi-automatic annotation method (see next section). In total, 67,033 nuclei and 526,348 cells were annotated for the nuclei detector and the PD-L1 detector respectively (see Supplementary Tables 1 and 2 for additional detail). Annotations were made using ASAP, an open-source in-house developed whole-slide image viewer.

### Deep learning models

**Figure 1:**
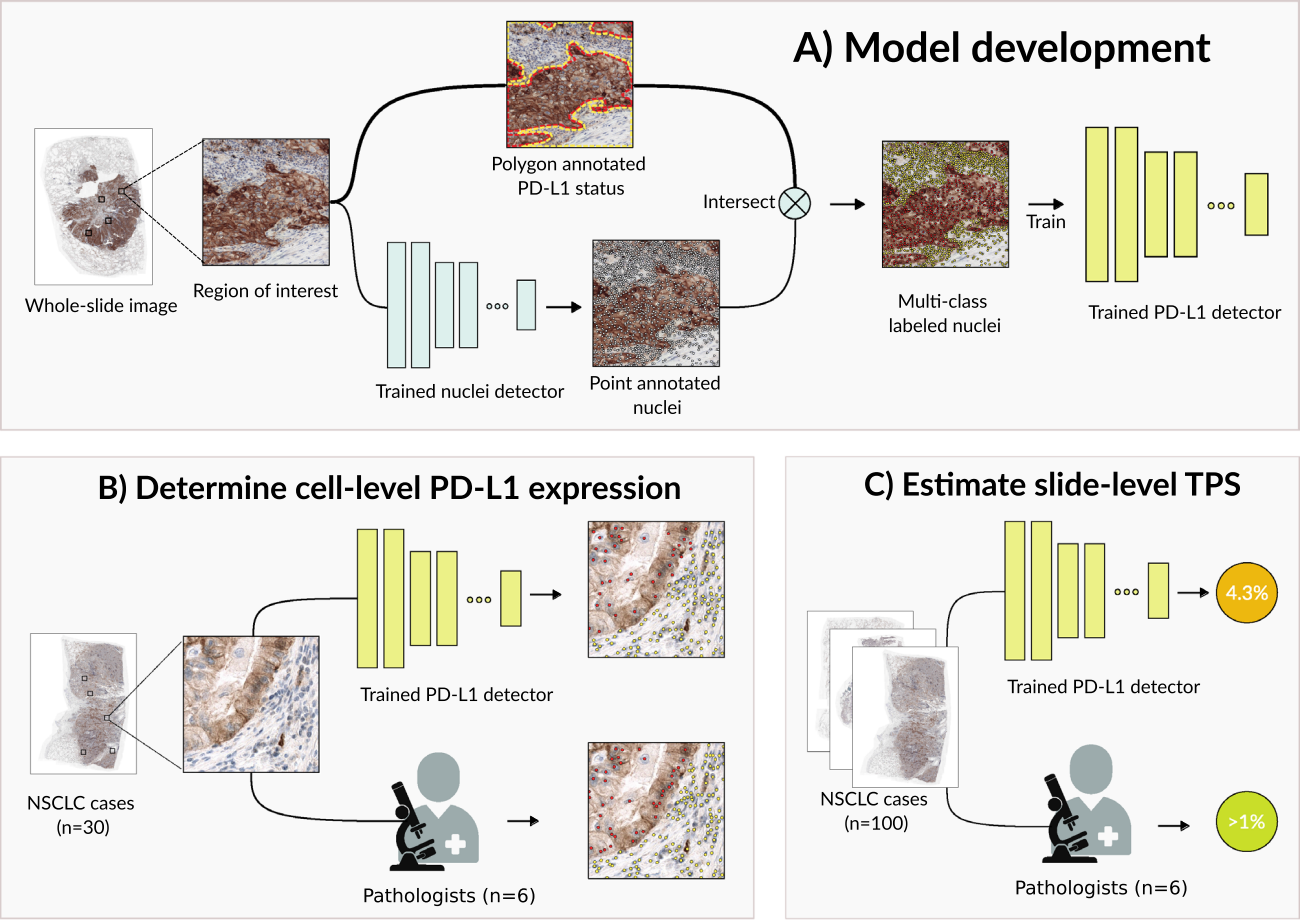
Overview of study design. In (**A**), the model development process is shown. First, a nuclei detector is trained using point annotated nuclei. Secondly, polygons are drawn in regions of homogeneous cell types and intersected with detected nuclei. The nuclei are given the label of the corresponding region, resulting in multi-class labeled nuclei usable for training a multi-class PD-L1 detector. The PD-L1 detector is validated by (**B**) correlating slide-level tumor proportion score as determined by the system with the visual estimate of pathologists and (**C**) comparing its detection output to manual cell-level annotations of pathologists.

The multi-class PD-L1 detector proposed in this paper was trained using fine-grained point annotations on a cell-per-cell basis. These are typically time intensive to produce, as an annotator must simultaneously determine both the cell’s type (classification) and its approximate center (localization). Therefore, we decoupled the localization aspect from the annotation task to allow annotators to focus on the more knowledge-intensive task of classifying cell type. We propose an efficient two-step annotation pipeline: first, experts make free-hand annotations in pre-selected regions of interest that label regions of homogeneous cell types; second, we developed a nuclei detector for the detection of nuclei centers in IHC; detected nuclei are intersected with the free-hand drawn polygons and then given the corresponding label. We show a schematic representation of using the nuclei detector’s output to generate a reference standard for the PD-L1 detector in Fig. 1A. The PD-L1 detector was developed to detect three cell types within NSCLC WSIs: PD-L1 positive tumor cells, PD-L1 negative tumor cells and an ‘other cells’ type. The ‘other cells’ type was used to make our algorithm applicable to WSIs, as these may contain many other structures than malignant cells.

We adopted the YOLOv5 convolutional neural network model architecture for both the nuclei and PD-L1 detectors, since it has strong computer vision performance with minimal required configuration. As YOLOv5 was originally designed for object detection with bounding boxes, we extend point annotations during training to bounding boxes of 20 by 20 μm, as determined by measuring the average size of cells within the dataset and adding a safety margin to this for cells with skewed aspect ratios. During inference we consider the center of the predicted bounding box as the detection location. We preprocess slides with a tissue segmentation network^21^ to limit analysis to tissue during whole-slide inference. Further details are given in the Supplementary materials and methods.

The nuclei detector and PD-L1 detector were developed using 35 and 39 slides respectively (taken from the RUMC and NEG datasets). The train/validation split for both algorithms is shown in Table 1 and was stratified in terms of NSCLC subtype and cell types, keeping the proportions identical between train and validation sets (Supplementary Table 3 shows the distribution of NSCLC subtypes per set). The validation set was used to monitor for overfitting during training (based on the classification, object and box loss of YOLOv5, see^22^ for details). The model weights that performed best on the validation set were used to compare the models to human readers using holdout test sets (see next section).

### Benchmarking of the algorithms against readers

Interobserver variability was measured amongst research assistants for nuclei detection and amongst expert pathologists for slide-level (i.e. TPS) and cell-level (i.e. ‘positive’ or ‘negative’ cells) determination of PD-L1 expression. These reader studies served as holdout test sets for the nuclei and PD-L1 detectors respectively (all slides were previously unseen by the algorithms). This allowed us to determine whether the algorithms performed within interobserver variability (the theoretical upper bound performance of an algorithm trained on answers from a single reader).

The nuclei detector was tested on ROIs within 12 resections from the RUMC dataset. Each ROI was exhaustively annotated for nuclei by four readers: three trained student assistants (L.M, M.D.A and D.Z) and one trained physician (G.S), labelled R_i_ through R_iv_.

For the PD-L1 detector, the interobserver variability for determining cell-level PD-L1 expression (Fig. 1B) was measured by inviting five expert pathologists and one trained physician to make manual annotations on ROIs within 30 cases (12 resections from the RUMC dataset, 9 resections from the NEG dataset and 9 biopsies from the NKI dataset). Each reader exhaustively point annotated ROIs for negative tumor cells, positive tumor and ‘other cells’. ROIs were approximately 150 by 150 μm (4–5 per case) and were selected by a pathologists (E.M.) The case series was read in two groups: RUMC cases were each annotated by 3 pathologists (A.E, E.M. and I.G, labeled P_1_ through P_3_) and the NEG and NKI cases were each annotated by 2 pathologists and the physician (B.A, C.B and G.S, labeled P_4_ through P_6_). The interobserver variability for determining slide-level PD-L1 expression (Fig. 1C) was measured by inviting six expert pathologists (E.M, I.G, A.E, K.G, S.V. and M.L.S., labeled P_a_ through P_f_) to score TPS in 100 cases (12 resections and 25 biopsies from the RUMC dataset, 38 resections from the NEG dataset and 25 biopsies from the NKI dataset). TPS was estimated as the percentage of viable tumor cells showing partial/complete circumferential membranous staining above background level in five percent increments. Of all readers, only P_d_ has not previously taken courses for the reading of PD-L1 stained IHC. In both experiments, cases were selected to represent a wide diversity of PD-L1 IHC (Supplementary Table 3), encompassing three PD-L1 monoclones (22C3 NKI, E1L3N and SP263). We refer to the RUMC and NEG cases as in-domain test cases, while the NKI cases served as out-of-domain test cases, meant to test the generalizability of the PD-L1 detector to a PD-L1 monoclonal antibody not included in its training set.

Cases were read via the online grand-challenge.org platform using the Reader Studies functionality (Supplementary Fig. 1). Whilst reading cases, readers could view the H&E and PD-L1 slide in parallel; whenever possible, this H&E slide was adjacent to the PD-L1 slide in terms of cutting order.

### Statistical analysis

To evaluate cell-level observer variability, we use a “hit criterion” based on the distance between predictions and a reference standard: a prediction is valid within an 8 μm radius of a reference point. This chosen radius was identical for all cell types and corresponds to the average diameter of tumor and immune cell nuclei in the dataset. If multiple predictions fall within this radius, only the closest one to the reference standard is considered a hit. Predictions labeled as the correct cell type are true positives, while others are false positives. Reference standards without associated predictions are false negatives, and predictions that do not hit any ground truth points are false positives. Using this criterion, the F1 score was computed, defined as the harmonic mean of precision and recall: 2*TP/(2*TP + FP + FN). We report the mean and class specific F1 score for all pairings of reader and PD-L1 detector.

We measured the agreement on scoring TPS in pairwise combinations of pathologists and the PD-L1 detector in two ways: in a continuous fashion via the intra-class correlation (ICC) using a two-way random effect model with absolute agreement [ICC(2,1)] and in a discretized fashion taking into account the clinically relevant cutoffs (< 1%, 1–49% and ≥ 50% TPS) via linear Cohen’s kappa.

95% confidence intervals were calculated by taking 1.96 standard deviations away from the mean for normally distributed metrics, as determined by a Shapiro-Wilks test. A significance level of 0.05 was chosen for all statistical tests.

## Results

### Agreement between readers and nuclei detector on localizing nuclei

**Figure 2:**
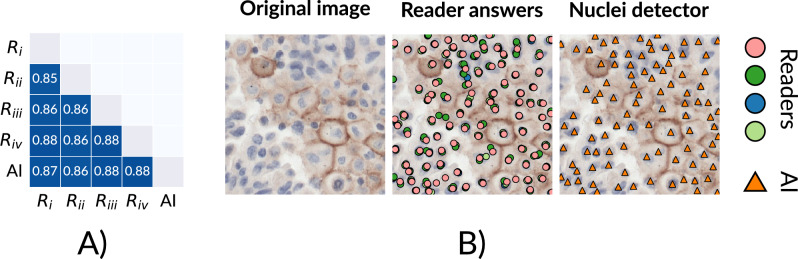
Validation of the nuclei detector. In (**A**), pairwise F1 scores are shown over cell predictions in the RUMC nuclei detection test set made by the nuclei detector and human readers. In (**B**), one ROI from the test set is shown with annotations by the readers and predictions from the nuclei detector overlaid on the PD-L1 immunohistochemistry. Annotations from the readers are shown as pink, light yellow, green and blue dots, while the AI predictions are shown as orange triangles.

The F1 scores per reader-reader/reader-algorithm pairing are shown in Fig. 2A and we show examples of predictions of the nuclei detector together with predictions of the three readers in Fig. 2B. Overall, the average reader-reader and reader-algorithm F1 scores were identical at 0.87. The lowest F1 score was measured between reader i and reader ii (0.85), while the highest F1 score was tied between i-iv, iii-iv, iii-AI and iv-AI (0.88). Visual inspection of the algorithmic predictions indicated that the nuclei detector performs equally well on PD-L1 negative and positive cells, as well as other cell types.

### Agreement between pathologists and PD-L1 detector on determining cell-level PD-L1 expression status

**Figure 3:**
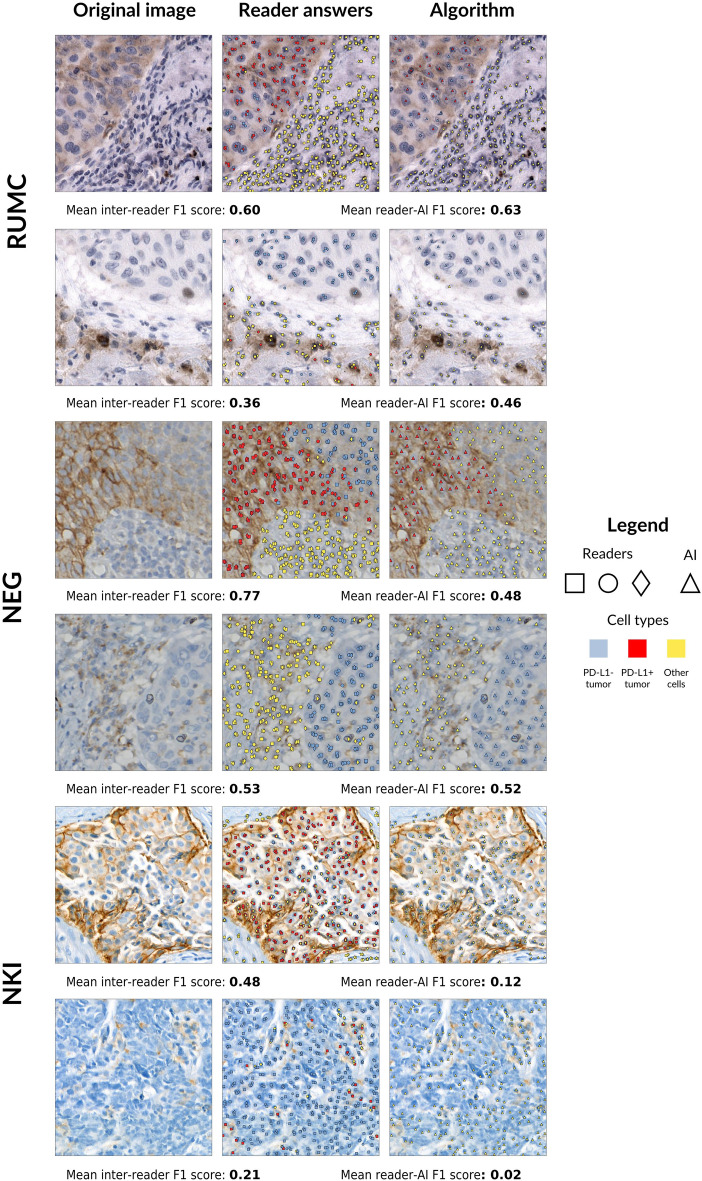
An overview of PD-L1 detector results as compared to the cell-level predictions of the readers. Each reader in the middle column has their own unique symbol (diamond, square or circle), while the PD-L1 detector results are always shown as triangles in the last column. The color of the symbol indicates the predicted class: blue for PD-L1 negative tumor cells, red for PD-L1 positive tumor cells, yellow for other cells. The mean F1 scores for all reader-reader (‘inter-reader’) and reader-AI pairs are shown per ROI underneath each row.

**Figure 4:**
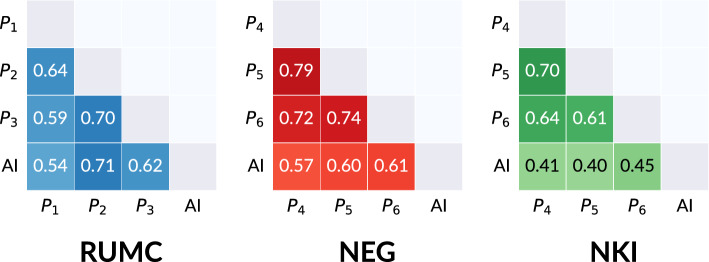
Pairwise F1 scores over the cases from the cell-level reader study, measuring the cell-level interobserver agreement of pathologists and AI on determining PD-L1 expression. The F1 scores are shown per clinical (*RUMC, NEG and NKI)* and are averaged over the three classes (PD-L1 negative/positive tumor cells and ‘other cells’). The F1 scores per class are reported in Supplementary Fig. 2.

We show examples of ROIs of all clinical centers alongside predictions of the six readers and the PD-L1 detector in Fig. 3. The F1 scores of all pairings are shown in Fig. 4, split per clinical center (we show F1 scores split per cell type in Supplementary Fig. 2). When comparing the mean reader-reader and reader-algorithm F1 scores, we find an overall F1 score of 0.68 versus 0.55. Considering each clinical center separately, we see similar performance on RUMC cases (0.64 versus 0.62), a mild performance drop for the PD-L1 detector on NEG cases (0.75 versus 0.59) and a substantial performance drop on the out-of-domain NKI cases (0.65 versus 0.42). The performance drop of the algorithm on NKI cases is most noticeable on the PD-L1 positive tumor cells: the mean F1 score for this cell type is 0.11. We show NKI cases in the last two rows of Fig. 3, where we can see that the PD-L1 detector falsely considers most cells as ‘other cells’.

### Agreement between pathologists and PD-L1 detector on determining slide-level TPS

**Figure 5:**
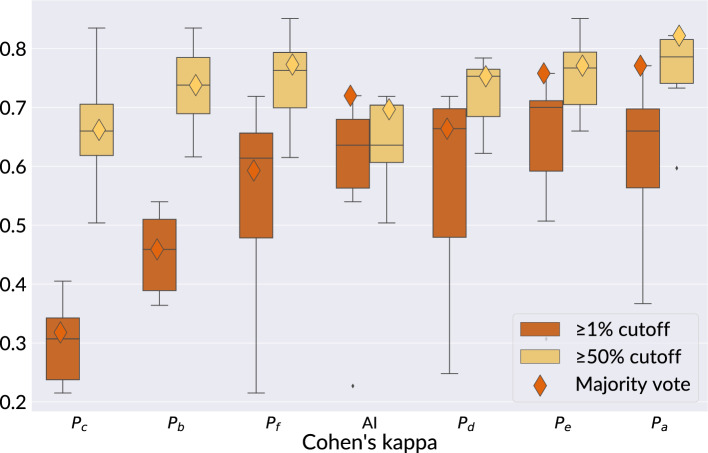
The distribution of the linear Cohen’s kappa agreement of readers with other readers for predicting the tumor proportion score (TPS) on n = 100 test set cases. For this analysis, agreement on TPS was considered solely at the 1% and at the 50% cutoff. The diamonds in each boxplot visualize the agreement of the reader with the majority vote of human readers for that particular cutoff. The boxplots of each reader are sorted on the x-axis according to the median agreement with the majority vote over both cutoffs.

When measured continuously, the mean reader-reader and reader-algorithm agreements on determining slide-level TPS were both good (ICC = 0.852 [95% CI 0.768–0.936] versus ICC = 0.796 [95% CI 0.694–0.898]). We show the ICC for all pairings in Supplementary Fig. 3. The mean difference between the median reader TPS and the PD-L1 detector TPS was − 4.70 (95% CI − 43.50 to 34.10), indicating that the PD-L1 detector systematically measured a lower TPS than the median score of the pathologists. We show the agreement between the median reader TPS and the PD-L1 detector for all cases in a Bland Altman plot in Supplementary Fig. 4 and show the mean difference per clinical center in Supplementary Table 4. When measured in a discretized fashion, the mean reader-reader kappa was 0.535 (95% CI 0.258–0.812) and the mean reader-AI kappa was 0.494 (95% CI 0.272–0.715) for all cutoffs together (< 1%, 1–49% and  ≥ 50%). The mean reader-AI kappas per clinical center for all cutoffs together are reported in Supplementary table 4. We show the distribution of kappa values per reader in Fig. 5 for the 1% cutoff and 50% cutoff alone and also plot the agreement versus the majority vote of the six readers (the kappa score of each reader compared to the majority vote was calculated excluding their own ratings). There, we see that for the difficult 1% cutoff, the PD-L1 detector has an agreement with the majority vote of 0.72, better than four of the six readers, while the maximum agreement with the majority vote was reached by P_a_ (0.77). For the 50% cutoff, the PD-L1 detector reaches an agreement with the majority vote of 0.697, only better than one pathologist, but it should be noted that all majority vote agreements are consistently good (0.662 to 0.822). Lastly, the PD-L1 has the lowest discrepancy between kappas on the 1% and 50% cutoffs.

## Discussion

In this work, we reported on a deep learning system designed for the automatic detection of PD-L1 positive and negative tumor cells in digital NSCLC histopathology images. These detections can be used subsequently for the automatic quantification of the tumor proportion score (TPS). The method consists of a single-stage PD-L1 cell detector, which was trained using a novel combination of a nuclei detector and polygon annotations to accelerate collection of the reference standard. We evaluate our system at (1) at cell-level by measuring the agreement with pathologists on estimating PD-L1 expression in individual cells and (2) at slide-level by measuring the agreement with pathologists on the clinically relevant task of scoring TPS.

The nuclei detector was validated against four human readers on an independent test set, where each reader annotated roughly 10,000 nuclei. There, the nuclei detector performed on par with the average reader (with an F1 score of 0.87), indicating the system performs within interobserver variability on tested data. The largest source of disagreement between readers and the nuclei detector arose in giant, multinucleated cells, where the nuclei detector predicted each distinct lobe as a nucleus. In addition, cells with a faint hematoxylin staining (possibly deeper within the z-stack of the tissue) were inconsistently annotated by both the nuclei detector and the readers.

In our cell-level reader study, the PD-L1 detector performs on par or slightly below the interobserver variability on the in-domain RUMC and NEG datasets, but underperforms compared to interobserver variability on the out-of-domain dataset of the NKI. Here, the F1 score on PD-L1 positive cells drops notably due to overprediction of ‘other cells’. We attribute this poor agreement to the fact NKI cases appear substantially different from the in-domain test cases, partially due to the custom 22C3 laboratory-developed test, which takes on an atypical red-brown color. Moreover, the tissue preparation of the NKI cases results in different tissue morphology, leading to further disparity between the in-domain and out-of-domain test sets. Lastly, there were no biopsies included in the training set of the PD-L1 detector, which may have withheld biopsy-unique morphological information from the PD-L1 detector.

Dealing with the domain-gap between the training set and test set of a model is an active research field in machine learning and there are multiple possible avenues to overcome the limited performance on the out-of-domain NKI dataset. First and foremost, the training set could be expanded with more multi-centric data, including biopsies (to account for possible morphological artifacts) and PD-L1 staining using the 22C3 antibody, optionally only including data scanned with the same scanner used at the NKI, to make the test set more comparable with the training set. Secondly, heavier data augmentation with augmentation techniques specifically tailored for IHC could be used. Moreover, style-transfer methods^23,24^ have been shown to close the domain gap. Lastly, stain normalization, although heavily used for applications using standard H&E WSIs, remains underused in immunohistochemistry applications and could be explored.

We show the confusion matrices that underlie the pairwise F1 scores of Fig. 4 in Supplementary Fig. 5. The most prevalent source of disagreement between readers was between PD-L1- tumor cells and ‘other cells’, most likely due to uncertainty on whether the cell is benign or malignant. The PD-L1 detector shows a similar pattern, but also frequently disagreed with readers on positive tumor cells, instead considering these as ‘other cells’, most likely due those cells being considered alveolar macrophages. It should be noted that the readers had access to the H&E stain while reading PD-L1 cases, while the PD-L1 detector did not, possibly making the morphological discrimination of macrophages easier. In the case that a reader or the PD-L1 detector predicted ‘other cells’ (such as stromal or inflammatory immune cells), there was almost always agreement with the other reader. Of note is the amount of times readers disagree amongst each other on the presence/absence of a cell (labeled ‘background’ in Supplementary Fig. 5), accounting for 22% of the disagreements on average. The PD-L1 detector has a significantly lower (p < 0.01) mean ‘background’ disagreement of 15% when comparing its predictions to annotations by readers, reflecting its tendency to localize cells more accurately. We associate this to the fact that when producing point annotation, the simultaneous focus on localization as well as classification of cells may overload readers, which highlights the efficacy of our semi-automatic annotation style, eliminating the localization aspect of annotating.

In our slide-level reader study, we find that the mean kappa of paired readers sits very close to the mean kappa of readers versus AI (0.535 versus 0.494 respectively, with overlapping confidence intervals), indicating the PD-L1 detector is able to quantify tumor proportion score at a level within interobserver variability. When considering the cutoffs separately, we find that the PD-L1 detector is more consistent around the cut offs than the readers, implying the algorithm can be valuable for cases that are difficult to interpret. This is further substantiated by the fact that the PD-L1 detector ranks third in terms of its kappa agreement with the majority vote on the 1% TPS cutoff.

We find lower kappa values compared to previous studies that measure interobserver variability of pathologists and the agreement of computer models and pathologists for (visually) estimating TPS (0.535 for interobserver and 0.494 for AI-pathologist agreement). Wu et al.^17^ measured the agreement between the majority vote of six pathologists and a deep learning system on 100 out-of-domain SP263 stained slides and found a Cohen’s kappa of 0.65 for TPS below 50% and 0.926 for TPS above 50%. Hondelink et al.^12^ correlated three pathologists to a proprietary algorithm on 139 in-domain 22C3 stained slides and found an overall Cohen’s kappa of 0.68. Choi et al.^15^ measured on 479 out-of-domain 22C3 stained cases, finding a mean Cohen’s kappa between 3 pathologists and their PD-L1 analyser of 0.67. All three studies also found comparable interobserver variability of visually estimating TPS by pathologists (K = 0.7–0.89, K = 0.61 and K = 0.739–0.871 respectively). We hypothesize that the unique multi-center, multi-stain setup of our reader study lowered both the agreement amongst pathologists and with the AI.

To our best knowledge, we provide for the first time a cellular benchmark for PD-L1 computer algorithms by measuring the interobserver variability of pathologists on determining cell-level PD-L1 expression. This precludes direct comparison to literature values. Only Choi et al.^15^ reports cellular F1 scores, but they do so only on PD-L1 negative and positive tumor cells within neoplastic regions (0.723/0.722, comparable to ours).

Our results are noteworthy because they indicate substantial interobserver variability, showing that the reference standard for PD-L1 related algorithms must be established very carefully, preferably using multiple readers fused via majority vote to promote robustness. Careful consideration must go into instructing readers on annotating and selecting readers that have sufficient experience in reading PD-L1 stains. The effect size of these variables could be the topic of further study, similar to Butter et al.^7^.

This study has some limitations. Firstly, we did not quantitatively measure the time gain of our semi-automatic annotation technique over other techniques. While the added value of the nuclei detector is most pronounced in areas of homogeneous cell types, time savings tend to be diminished in areas of heterogeneous cell types. Secondly, for the cell-level F1 score metric, we cannot distinguish between instances where readers overlooked a cell versus cases where they believed that a particular shape did not represent a cell, leading to potentially lower mean F1 scores, especially for the readers as their localization accuracy was noticeably worse than that of the PD-L1 detector. Thirdly, we did not measure the intraobserver variability of readers for the cell-level reader study. Fourthly, there was a substantial class imbalance for the PD-L1 detector training set, as PD-L1 positive tumor cells only accounted for 8% of the annotations. This may have contributed to the reader-AI agreement being consistently lower for positive tumor cells. Finally, pathologists had access to morphological information from H&E slides in the TPS reader studies, which was not available to our AI algorithm. Although not required for AI to address the task of multi-class cell detection, this factor might play a role in some of the differences observed in TPS scores between experts and AI.

In conclusion, we developed a deep learning system for the automatic detection of PD-L1 positive and negative tumor cells. While doing so, we performed reader studies and provided the first interobserver cell-level analysis on PD-L1, which we used to measure and report the agreement amongst readers and AI-reader agreement on a cellular basis and at TPS level. Future work should focus on improving the model to generalize better to unseen antibodies and analyze the predictive value of the automatically quantified TPS for ICI treatment response as compared to pathologists (similar to Kapil et al.^25^). Lastly, the model’s cell-level predictions could be used to develop (spatial) biomarkers that go beyond the predictive value of TPS.

### Supplementary Information

Supplementary Information.

## Data Availability

The RUMC dataset is available from the corresponding author on reasonable request. The NEG and NKI datasets are not available as these were shared with the authors by third parties under licenses that preclude further dissemination. The authors publicly release the nuclei detector and the PD-L1 for model inference on Grand Challenge: https://grand-challenge.org/algorithms/tumor-proportion-score-in-non-small-cell-lung-canc/. https://grand-challenge.org/algorithms/nuclei-detection-in-immunohistochemistry/.
