# Supplementary material for: Comparing deep learning and pathologist quantification of cell-level PD-L1 expression in non-small cell lung cancer whole-slide images
Source: Sci Rep. 2024 Mar 26;14:7136. doi: 10.1038/s41598-024-57067-1 (PMC10965947; doi:10.1038/s41598-024-57067-1)
Supplement: Supplementary file 1 — Supplementary Information. [file 41598_2024_57067_MOESM1_ESM.pdf]

Comparing deep learning and pathologist quantification of cell-level PD-L1 expression in non-small cell lung cancer whole-slide images

Supplementary material

1. Supplementary materials & methods

Datasets

The RUMC dataset was stained for PD-L1 with the E1L3N assay (Cell Signaling Technology, USA) using a Labvision 480 autostainer (Thermoscientific, USA). Resections were scanned with a PANNORAMIC 250 scanner and biopsies were scanned with a PANNORAMIC 1000 scanner (3DHistech, Hungary), both at a pixel resolution of 0.25 micrometers.

For the NEG dataset, surgical resection specimens were stained with the SP263 assay (Roche, Switzerland). For the TMA cores, 10 were stained with the 22C3 assay (Agilent, USA), referred to as 22C3 Dako in this text, and 6 were stained with the SP263 assay. SP263 slides were stained using the Ventana Benchmark Ultra platform (Roche) while 22C3 Dako slides were stained using the 22C3 Dako Link-48 autostainer system (Agilent). All slides were scanned using a Ventana iScan HT scanner (Roche) at a pixel resolution of 0.5 micrometers.

The NKI dataset was stained using a 22C3 laboratory-developed test (referred to as 22C3 NKI) using the Ventana Benchmark Ultra platform and were scanned using a PANNORAMIC 1000 scanner at a pixel resolution of 0.25 micrometers.

Deep learning models

We use the YOLOv5 object detection model as it offers strong off-the-shelf computer vision performance with minimal required configuration. The YOLOv5 model is a one-stage detector, consisting of a backbone (CSP-Darknet53 [1]) that functions as a feature extractor, a neck (SPPF [2] and PAN [3]) that aggregates the features and finally a head (YOLOv3 [4]) for generating the prediction.

Our hyperparameter settings were identical between the nuclei and the PD-L1 detector and were tweaked minimally from the stock configuration of YOLOv5: i) we remove the mosaic and translation augmentations to preserve a more realistic appearance of histopathology images, ii) we add the HSV-light augmentations as described by Tellez et al. [5], iii) we increase the maximal number of detections to

1.500 during non-maximum suppression postprocessing,

YOLOv5 was trained using 512 x 512 sized patches randomly sampled at a resolution of 0.5 micrometers per pixel from annotated ROIs in our training set. All model predictions were thresholded at a confidence value of 0.5. We train the YOLOv5 medium model architecture from the pretrained COCO checkpoint.

2. Supplementary figures

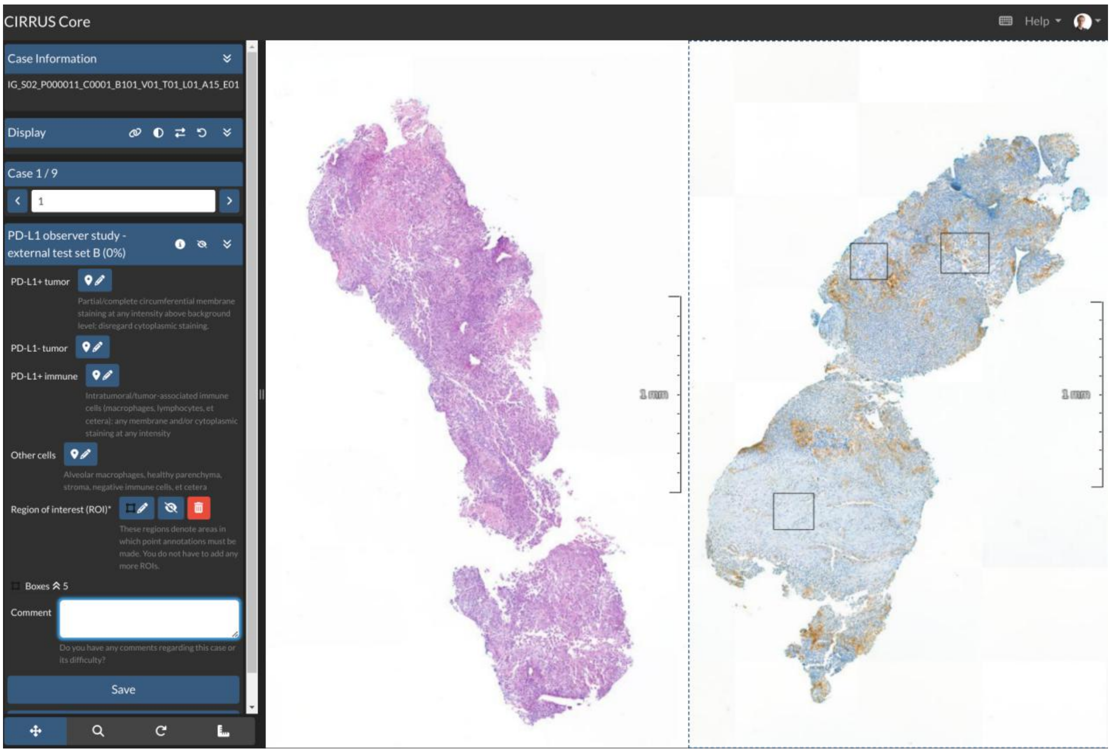

Supplementary figure 1: An example case from the web platform used by pathologists to read cases for tumor proportion score and cell-level PD-L1 expression. The invited pathologists could simultaneously view a (paired) H&E and PD-L1 stained slide and zoom in independently. In this example, three regions of interest are visible as the dark boxes, in which pathologists annotated PD-L1 negative/positive tumor cells and ‘other cells’.

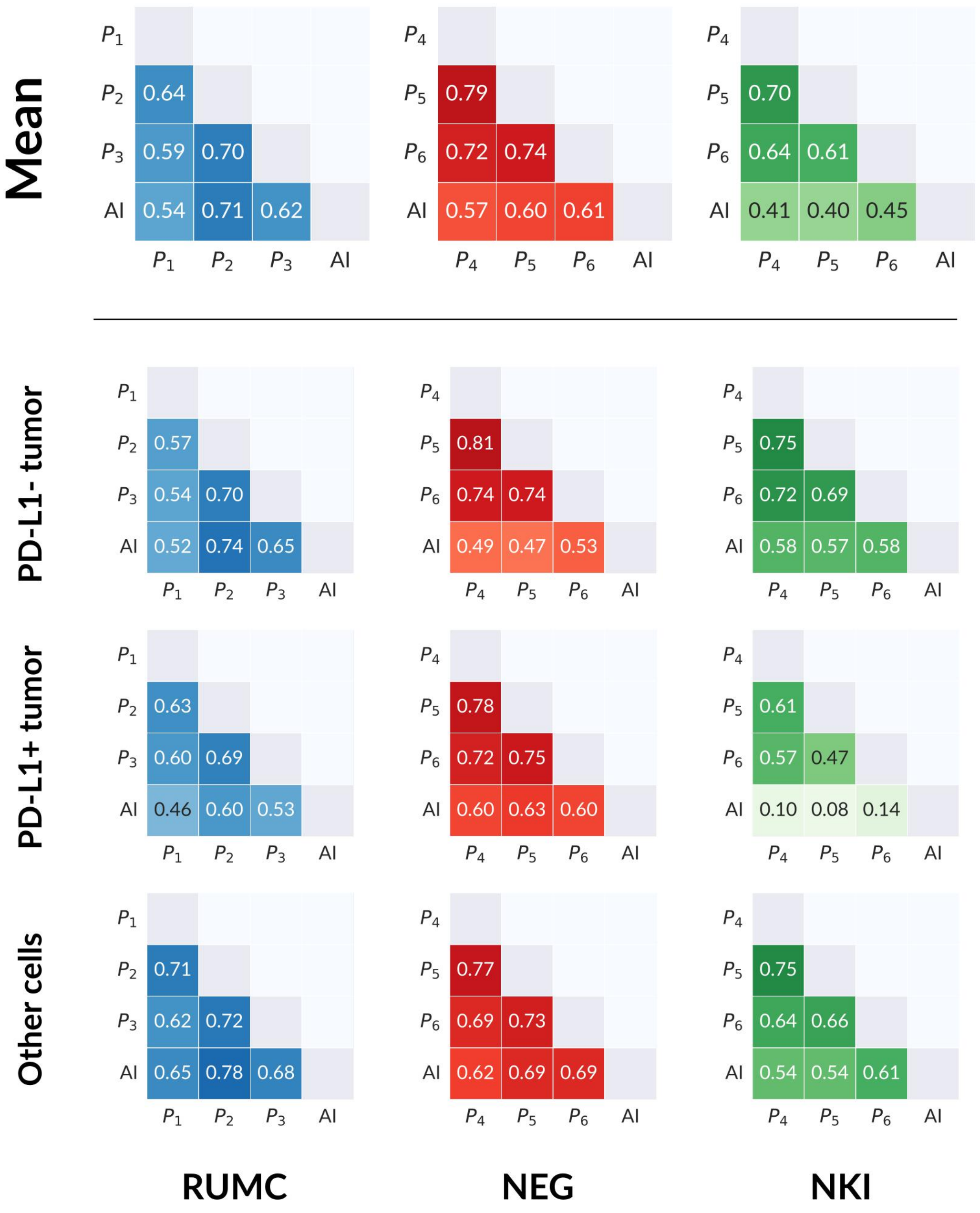

Supplementary figure 2: Pairwise F1 scores over the cases from the cell-level reader study, measuring the cell-level inter-rater agreement of pathologists and AI on determining PD-L1 expression. The F1 scores are shown per clinical (*RUMC, NEG and NKI)* and per class (PD-L1- tumor cells, PD-L1+ tumor cells and 'other cells').

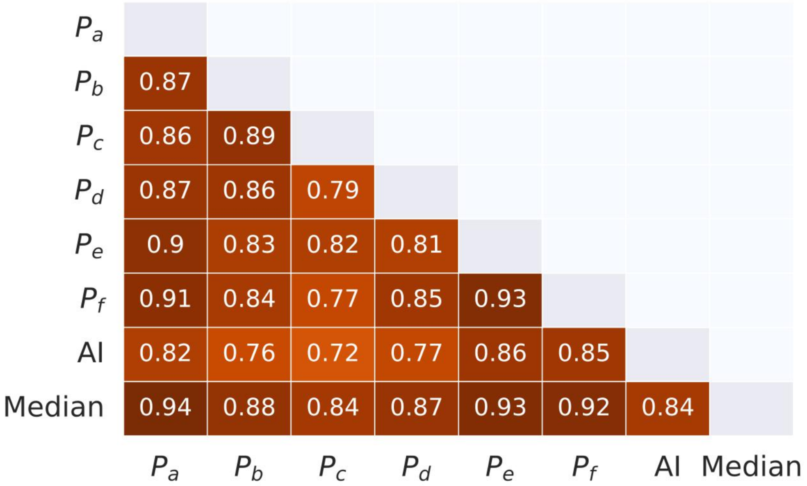

Supplementary figure 3: Intra-class correlation (ICC) of the TPS as estimated by all pairings of readers and PD-L1 detector on the hundred cases of the slide-level reader study.

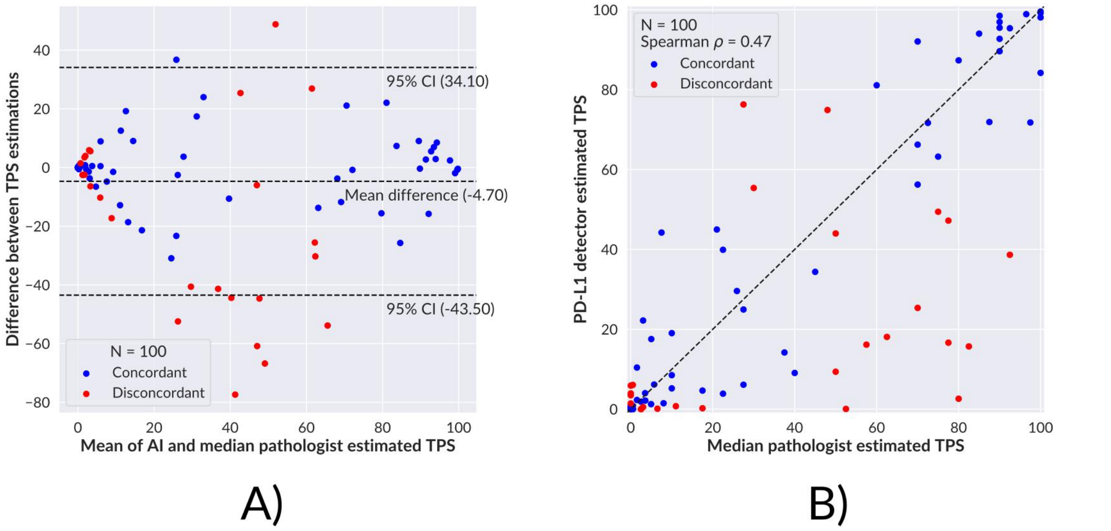

Supplementary figure 4: The graphed relationships between the TPS as estimated by the median pathologist and the PD-L1 detector for the hundred cases of the slide-level reader study. A) Bland-Altman plot showing the mean between the AI-determined TPS and the median pathologist estimated TPS versus their differences. B) Scatter plots showing the median pathologist estimated TPS versus the PD-L1 detector estimated TPS.

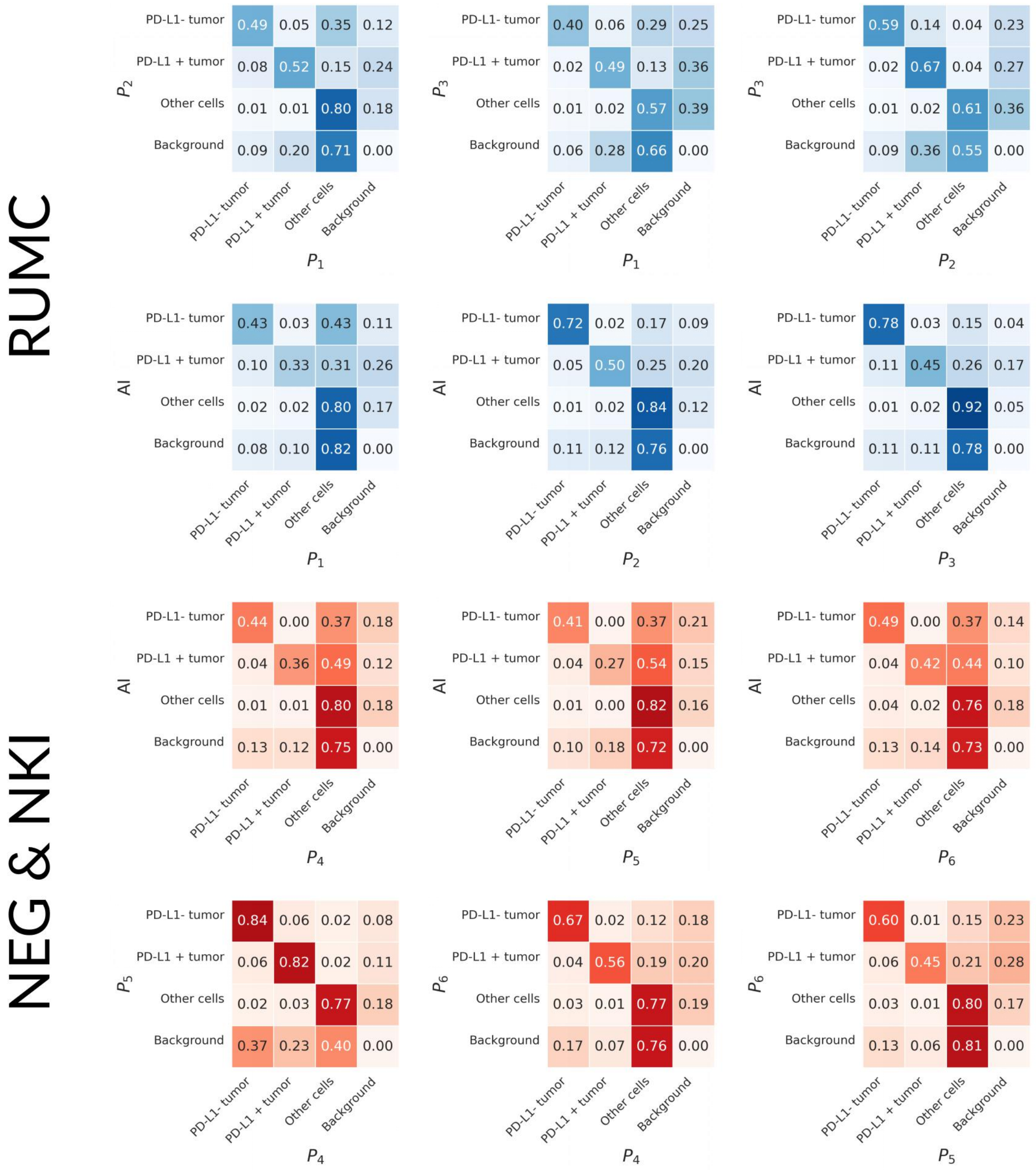

Supplementary figure 5: Confusion matrices measuring the (dis)agreement of every reader-reader or reader-AI pair. The diagonal entries of each matrix represent the percentage of times reader 1 (y-axis) agreed with reader 2 (x-axis) on that particular cell type, while the off-diagonal entries represent which percentage of times reader 2 predicted a different cell type instead. The last row of the matrices represent instances where predictions of reader 1 did not have a corresponding prediction of reader 2 within an 8 micron margin. The matrices are normalized in the rows.

3. Supplementary tables

Dataset	#Slides	#Rois	#Annotated nuclei (train/val)
RUMC	31	109	16,984 / 13,617
NEG	4	5	24,476 / 11,756
Sum	35	114	41,660 / 25,373

Supplementary table 1: Annotations for the development of the nuclei detector.

Dataset	#Patients	#Rois	#PD-L1- tumor (train/val)	#PD-L1+ tumor (train/val)	#Other cells (train/val)	#Adeno (train/val)	#Squamous cell (train/val)	#Large cell (train/val)
RUMC	23	168	76,923 / 7,841	32,939 / 1,652	259,327 / 57,188	11/1	8/3	0/0
NEG	16	80	19,777 / 18,815	4,220 / 1,428	21,768 / 24,470	7/4	0/0	3/2
Sum	39	184	96,700 / 2,6656	37,159 / 3,080	281,905 / 81,658	23	11	5

Supplementary table 2: Annotations for development of PD-L1 detector.

			Subtypes		
**Algorithm**	**Subset**	**Total**	**LUAD**	**LUSC**	**Other**
Nuclei detector	Training	21	14	7	0
	Validation	14	6	8	0
	Test	12	6	6	0
PD-L1 detector	Training	29	18	8	3
	Validation	10	5	3	2
	Cell-level test set	30	10	12	8
	Slide-level test set	100	37	37	26

Supplementary table 3: Distribution of NSCLC subtypes and tissue types per algorithm and subset.

Dataset	N	Mean AI-reader difference (95% CI)	Mean AI-reader kappa for all cutoffs (95% CI)
RUMC	37	-11.1 (-47.94 - 25.74)	0.382 (-0.040 - 0.804)
NEG	38	5.52 (-19.96 - 31.01)	0.600 (0.269 - 0.931)
NKI	25	-10.77 (-55.94 - 34.39)	0.381 (0.070 - 0.692)
All	100	-4.7 (-43.50 - 34.10)	0.494 (0.247 - 0.740)

Supplementary table 4: Agreement metrics between the pathologists and the PD-L1 detector. We show the mean difference between the median TPS score of pathologists and the PD-L1 detector, in addition to the mean kappas of the pathologists and the PD-L1 detector (for all cutoffs together). Values are shown for all cases together and per center.

4. Supplementary references

1. Wang, C. *et al.* CSPNet: A new backbone that can enhance learning capability of cnn. Proceedings of the IEEE Conference on Computer Vision and Pattern Recognition Worksho, 2, 7 (2020)
2. He, K. *et al.* Spatial pyramid pooling in deep convolutional networks for visual recognition. IEEE Transactions on Pattern Analysis and Machine Intelligence, 37(9):1904–1916, . 2, 4, 7 (2015).
3. Liu. S. *et al.* Path aggregation network for instance segmentation. Proceedings of the IEEE Conference on Computer Vision and Pattern Recognition (CVPR), pages 8759–8768, 1, 2, 7 (2018).
4. Redmon, J. and Farhadi, A. "Yolov3: An incremental improvement." arXiv preprint arXiv:1804.02767 (2018).
5. Tellez, D. *et al.* Quantifying the effects of data augmentation and stain color normalization in convolutional neural networks for computational pathology. *Med. Image Anal.* **58**, 101544 (2019).
